# Supplementary material for: Shedding light on the circadian clock of the threespine stickleback
Source: J Exp Biol. 2021 Dec 17;224(24):jeb242970. doi: 10.1242/jeb.242970 (PMC8729910; doi:10.1242/jeb.242970)
Supplement: Supplementary information [file jexbio-224-242970-s1.pdf]

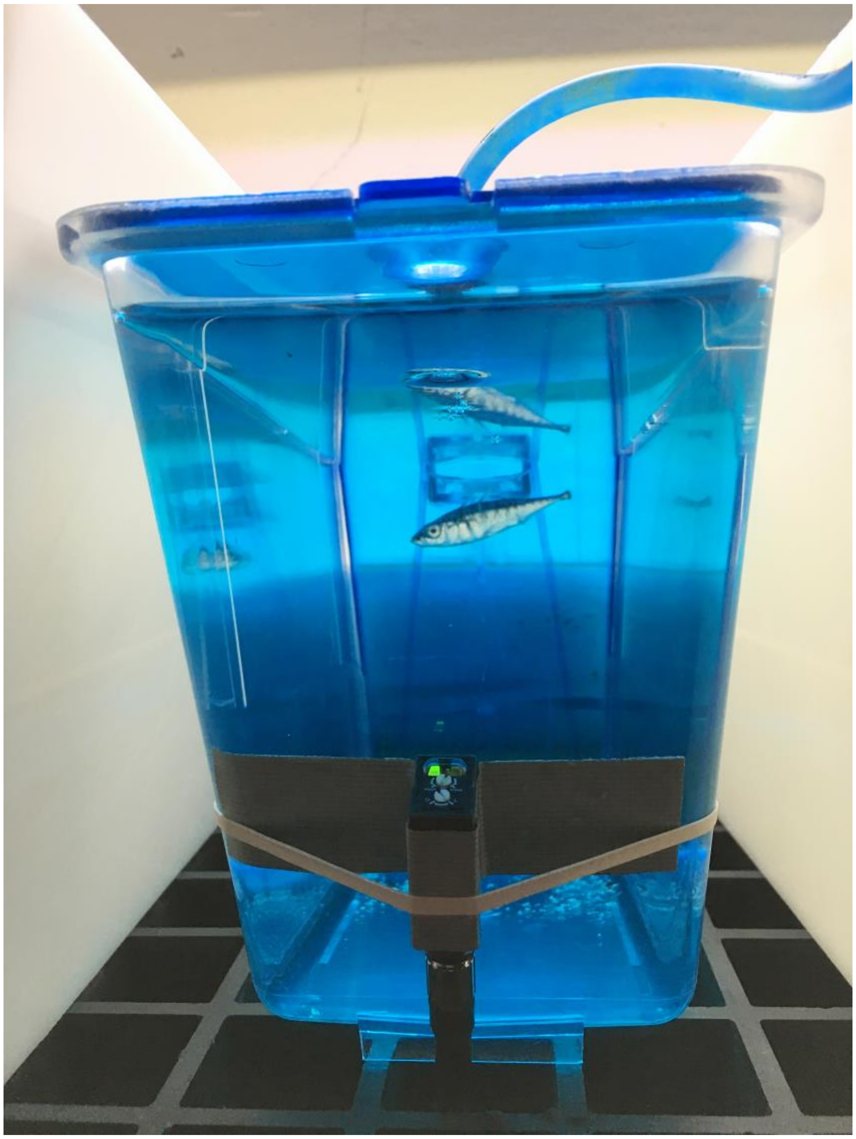

Fig. S1. Position of the infrared photoelectric sensor on an experimental tank. Each experimental tank was equipped with one infrared photoelectric sensor placed in the lower third of the front wall. Every interruption of the infrared light beam by the fish was counted as one movement.

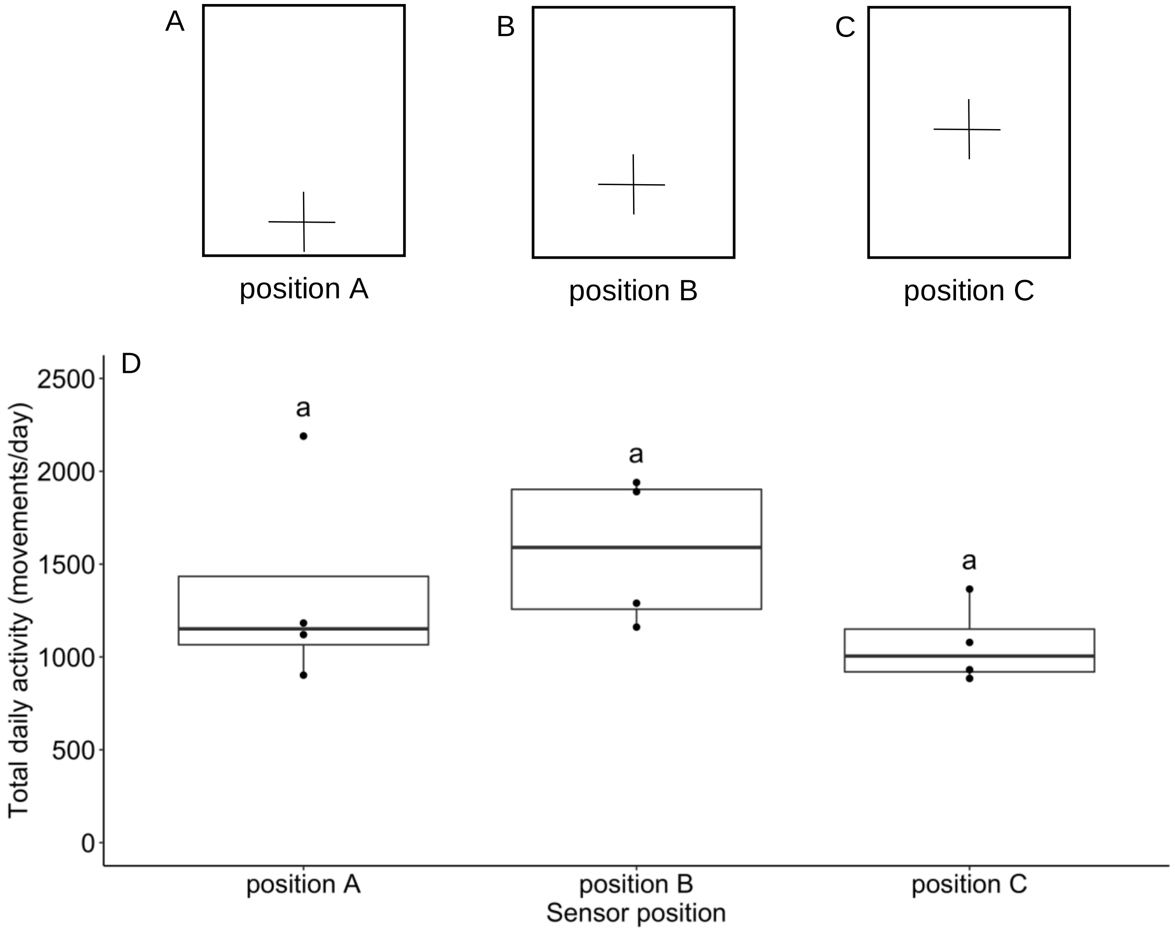

Fig. S2. Sensors position optimization. (A, B, C) In order to determine in which position the sensors detect the most movements, we did a pilot study using 12 fish. We put each fish in an experimental tank equipped with a sensor that was either placed at the very bottom (position A), in the lower third (position B) or in the middle of the front wall (position C), so that there were 4 fish per position (n=4/position). We monitored locomotor activity for 8 full days under a 12 h light:12 h dark cycle. The rectangle and the cross represent the front wall of the tank and the position of the sensor, respectively. (D) Average of the total daily activity (movements/day, average for 8 days) depending on the position of the sensor. Although there is no significant difference between positions (as indicated by the letter “a”, one-way ANOVA, $p>0.05$), the sensors in position B detect slightly more movements. We thus chose position B for our experiments (see Fig. S1). The black line in the middle of each boxplot indicates the median and each dot represents an individual. Note that we also divided this analysis into light and dark phases to test if the lack of difference persisted (analysis not shown). There was no difference in activity (movements/12-h) between the three positions for both the light phase (one-way ANOVA, $p>0.05$) and the dark phase (one-way ANOVA, $p>0.05$).

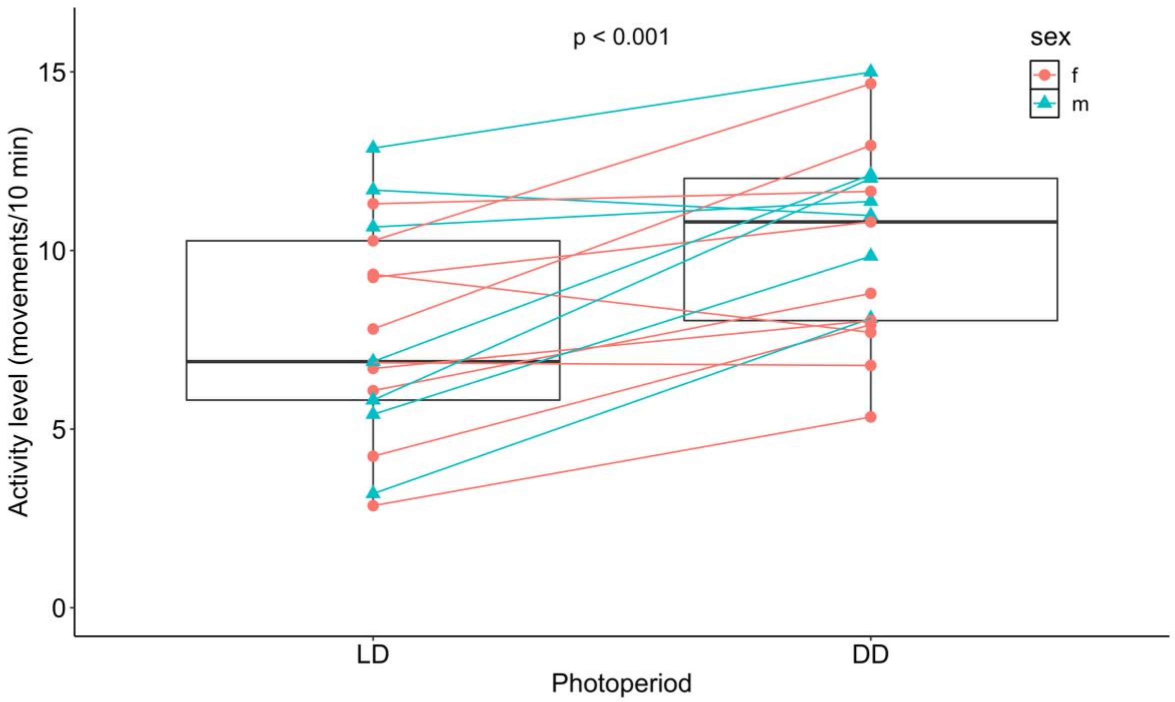

Fig. S3. Sticklebacks are less active during the light phase in LD than during the subjective light phase in DD. Average activity level (movements/10 min) for each individual during the light phase of a 12 h light:12 h dark cycle (LD, average for 8 days) and during the subjective light phase in constant darkness (DD, average for 10 days). Paired t-test, $p<0.001$, $n=17$.
